# *DeepCRISPR*: optimized CRISPR guide RNA design by deep learning

**DOI:** 10.1186/s13059-018-1459-4

**Published:** 2018-06-26

**Authors:** Guohui Chuai, Hanhui Ma, Jifang Yan, Ming Chen, Nanfang Hong, Dongyu Xue, Chi Zhou, Chenyu Zhu, Ke Chen, Bin Duan, Feng Gu, Sheng Qu, Deshuang Huang, Jia Wei, Qi Liu

**Affiliations:** 1 Department of Endocrinology & Metabolism, Shanghai Tenth People’s Hospital, Tongji University, Shanghai, 20009 China; 2 Bioinformatics Department, School of Life Sciences and Technology, Tongji University, Shanghai, 20009 China; 3 Machine Learning & Systems Biology Lab, School of Electronics and Information Engineering, Tongji University, Shanghai, 201804 China; 4 R&D Information, Innovation Center China, AstraZeneca, 199 Liangjing Road, Shanghai, 201203 China; 5 School of Life Science and Technology, ShanghaiTech University, Shanghai, China; 6 State Key Laboratory Cultivation Base and Key Laboratory of Vision Science, Ministry of Health and Zhejiang Provincial Key Laboratory of Ophthalmology and Optometry, School of Ophthalmology and Optometry, Eye Hospital, Wenzhou Medical University, Wenzhou, Zhejiang, 325027 China

**Keywords:** CRISPR system, Gene knockout, Deep learning, On-targets, Off-targets

## Abstract

A major challenge for effective application of CRISPR systems is to accurately predict the single guide RNA (sgRNA) on-target knockout efficacy and off-target profile, which would facilitate the optimized design of sgRNAs with high sensitivity and specificity. Here we present *DeepCRISPR*, a comprehensive computational platform to unify sgRNA on-target and off-target site prediction into one framework with deep learning, surpassing available state-of-the-art in silico tools. In addition, *DeepCRISPR* fully automates the identification of sequence and epigenetic features that may affect sgRNA knockout efficacy in a data-driven manner. *DeepCRISPR* is available at http://www.deepcrispr.net/.

**Electronic supplementary material:**

The online version of this article (10.1186/s13059-018-1459-4) contains supplementary material, which is available to authorized users.

## Background

CRISPR-based gene knockout is widely implemented in various cell types and organisms. In this system, a single-guide RNA (sgRNA) guides Cas9 proteins to specific genomic targets. Recognition and cleavage occur via complementarity of a 20-nucleotide (nt) sequence within the sgRNA to the genomic target, i.e., the on-target, upstream of a protospacer adjacent motif (PAM) at its 3′ end [1]. Extensive studies have also demonstrated that multiple mismatches as well as DNA or RNA bulges can be tolerated, resulting in cleavage of unintended genomic sites, termed off-targets [2]. Such a CRISPR-Cas9 endonuclease system permits genome editing at nucleotide resolution [3, 4], while a major challenge for its effective application is to accurately predict the sgRNA on-target knockout efficacy and off-target (OT) profile beforehand. Accurate prediction would facilitate the optimized design of sgRNAs by maximizing their on-target efficacy (high sensitivity) and minimizing their off-target effects (high specificity) [1, 2, 5–7].

Various sgRNA design rules and tools have been developed for sgRNA on-target identification and efficacy prediction. These methods are categorized into three types: (1) alignment-based, where the sgRNAs are aligned from the given genome purely by locating the PAM (*CasFinder* [8], etc.); (2) hypothesis-driven, where the sgRNA knockout efficacies are scored empirically by considering the impact of genome context factors (*E-CRISP* [9], *CRISPR* [6], *CHOPCHOP* [10], *GuideScan* [11], etc.); and (3) learning-based, where the sgRNA knockout efficacies are predicted from a training model by considering different features (*sgRNA Designer* [2], *SSC* [5], *sgRNA Scorer* [12, 13], *CRISPRscan* [14], etc.). A benchmark study indicated that the latter two types of tools generally perform better than the alignment-based tool, although the predictions do not scale well in different cell types [15, 16]. Novel computational methods as well as comprehensive exploration of DNA sequence and epigenetic features affecting sgRNA knockout efficacy are required [7, 16].

Off-targets are proven to occur in CRISPR system [2, 6, 17]. Although sgRNA-guided Cas9 cutting at a particular site does not necessarily lead to functional consequences (such as an in-frame shift mutation) [18], how to accurately and quantitatively detect or predict off-target cleavage sites is still an important issue and remains challenging [19]. Most existing tools use simple sequence alignment with different nucleotide mismatches to exhaustively search for off-target sites [20]. A few tools predict cleavage efficacy at the mismatched locus by designing off-target scores (for example, *CFD* score [2], *MIT* score [6, 16, 21], etc.). Their prediction results were compared with data generated by whole sgRNA off-target cleavage detection techniques like *GUIDE-seq* [22], *Digenome-seq* [16, 23, 24], High-throughput genome-wide translocation sequencing (*HTGTS*) [25], direct in situ breaks labeling sequencing (*BLESS*) [26], and integration-deficient lentiviral vector capture (*IDLV*) [27]. These are essentially hypothesis-based methods that use empirically defined off-target criteria to identify off-target sites. Effective learning-based prediction of the whole genome off-target profile is needed.

Currently, building a learning model for sgRNA efficacy prediction faces several obstacles: (1) data heterogeneity issues where effective integration is required for data from different cell types and experimental platforms. (2) data sparsity issues where the labeled sample size, i.e., the amount of sgRNAs with known efficacies, is relatively small and experimentally expensive to collect—insufficiently labeled data makes the current learning models inefficient; (3) data imbalance issues in off-target site prediction—the number of true off-target cleavage sites recognized by whole-genome off-target detection techniques is small among all the possible nucleotide mismatch loci; (4) the leading sequence and epigenetic features affecting sgRNA efficacy remain unclear and await further exploration [5].

Recently, several studies have tried sophisticated learning models for on-target or off-target prediction [28–30], but none of them have addressed these issues thoroughly. In our study, we present a novel and powerful deep learning framework [31–33] to simultaneously predict sgRNA on-target knockout efficacy and a whole-genome off-target cleavage profile that competes favorably with the available state-of-the-art tools. Our approach, called *DeepCRISPR*, is based on a carefully designed hybrid deep neural network for model training and prediction. To the best of our knowledge, this is the most comprehensive computational platform available to unify on-target and off-target site prediction into one framework with deep learning. We applied a *deep unsupervised representation learning* technique [32, 34] to automatically learn the underlying representation of sgRNAs using a complete set of genome-wide unlabeled sgRNAs. The learned model was further tuned by a supervised deep neural network using the existing labeled sgRNAs. We point out that pre-training of huge amounts of unlabeled sgRNAs can be used to boost the model prediction, which has never been studied in traditional sgRNA efficacy prediction. Together, *DeepCRISPR* addresses the above challenges with the following advantages: (1) by considering the epigenetic information in different cell types, it represents different DNA regions from different cell types in a unified feature space and integrates the data from different experiments and cell types. Although *DeepCRISPR* is trained on limited cell type data, we validated that it has a generally good prediction ability when adapting to new cell types. (2) It learns from billions of genome-wide unlabeled sgRNAs to automatically derive a “parent network”, thus generating a high-level feature representation simultaneously for sgRNA on-target and off-target design. In this way, *DeepCRISPR* optimizes sgRNA design for both coding and non-coding regions by considering the unsupervised pre-training of genome-wide sgRNA sequences from these regions. The models with and without pre-training were compared and the superiority of unsupervised pre-training was validated. (3) It applies a specific data augmentation technique to generate novel sgRNAs with biologically meaningful labels, thus increasing the labeled training size in sgRNA on-target site prediction. We further validated that such data augmentation indeed improves the prediction performance and makes the training model robust. (4) It further fine-tunes the parent network using the labeled sgRNA data, which helps to boost the prediction performance on limited labeled samples. (5) It integrates an efficient bootstrapping sampling algorithm with the training procedure, dramatically alleviating the data imbalance issue in off-target site prediction. (6) Finally, it fully automates the identification of sequence and epigenetic features. The model learns which features are important in optimized sgRNA design using limited training samples. The identified features can be used for optimized sgRNA design. This helps to decipher the CRISPR on-target and off-target mechanisms in a much more efficient data-driven manner.

*DeepCRISPR* is available at http://www.deepcrispr.net/. The command line code is also available at https://github.com/bm2-lab/DeepCRISPR and https://zenodo.org/record/1246320. The current version of *DeepCRISPR* focuses on conventional NGG-based sgRNA design for *SpCas9* in humans*.* It can be easily extended to other Cas9 species or variants and other species. Its on-target and off-target site prediction performances were compared with the available state-of-the-art tools.

## Results

### Training *DeepCRISPR* for sgRNA on-target and off-target site prediction

#### Deep unsupervised learning for sgRNA representation

**Fig. 1:**
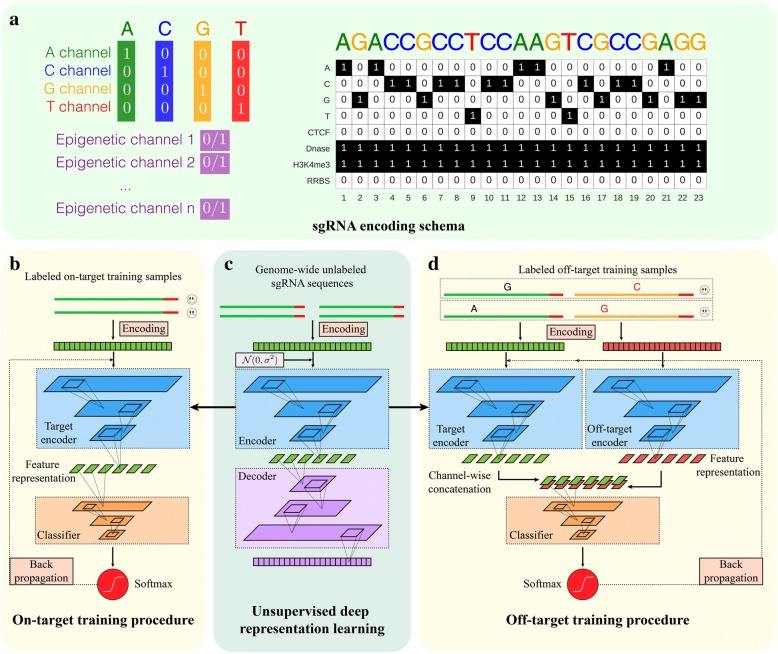
Implementation details of *DeepCRISPR.* **a** sgRNA encoding schema. For a DNA region, the nucleotide sequence is represented by four channels, i.e., the A-channel, C-channel, G-channel, and T-channel, and each epigenetic feature is considered as one channel. **b** Training details of *DeepCRISPR* for sgRNA on-target efficacy prediction. The *Softmax* and *Identity* functions correspond to classification and regression models, respectively. **c** Unsupervised deep representation learning based on billions of genome-wide sgRNA sequences. **d** Training details of *DeepCRISPR* for sgRNA off-target profile prediction. The *Softmax* and *Identity* functions correspond to classification and regression models, respectively

The first input of *DeepCRISPR* is the complete set of 20-bp sgRNA sequences with an NGG PAM across the human genome. We extracted all the sgRNA sequences with an NGG PAM from human coding and non-coding regions. These data account for ~ 0.68 billion sgRNA sequences with different epigenetic information curated from 13 human cell types (see the “sgRNA encoding with genome and epigenetic features” section). They serve as a large-scale unlabeled sgRNA data source for the following pre-training procedure to derive an efficient feature representation of sgRNA. The whole data collection and preprocessing were achieved by using a *SPARK*-based large-scale data processing architecture with *graphic processing unit* (*GPU*) acceleration. Each sgRNA is initially encoded with its sequence and epigenetic information (see the “sgRNA encoding with genome and epigenetic features” section). Then with these unlabeled sgRNA sequences in hand, we use a *deep unsupervised representation learning* strategy to train a *deep convolutionary denosing neural network (DCDNN)*-based autoencoder [35] to automatically learn the underlying meaningful representation of sgRNAs in an unsupervised manner [34] (Fig. 1c; see the “DCDNN-based autoencoder for representation learning” section). Such a de-noising strategy helps to train the autoencoder to robustly tolerate the noise in the huge amount of sgRNA data. The intuitive rationale to use autoencoder is that the unlabeled data with encoding and decoding can be used to learn an efficient feature representation. Such a learned feature representation will be fitted to the following model building. The network trained at this step is termed an unsupervised, pre-trained parent network for further analysis.

#### A fine-tuned hybrid deep neural network for sgRNA on-target knockout efficacy prediction

We next generated a hybrid deep neural network for sgRNA on-target knockout efficacy prediction, comprising two parts. The first part is the former pre-trained *DCDNN*-based network (parent network), the output of which is used as the input for a *convolutionary neural network* (*CNN*; Fig. 1b, c; see the “*CNN* model with pre-training based fine-tuning” section). The whole hybrid neural network was then trained based on the labeled data, i.e., the collected sgRNAs with known on-target knockout efficacies. The training procedure not only learned the weights for the *CNN*-based network, but also fine-tuned the weights of the parent network. Therefore, this strategy uses limited labeled data to tune the original pre-training network weights and it is expected to boost the prediction accuracy (Fig. 1b, c; see the “*CNN* model with pre-training based fine-tuning” section). In our study, the labeled sgRNA dataset contains ~ 0.2 million sgRNAs with known knockout efficacies. This dataset was generated from ~ 15,000 sgRNAs across 1071 genes with known knockout efficacies in a data augmentation manner (see the “On-target data sources” section), like that used for image data processing (see the “Data augmentation for on-target dataset” section). The final tuned weights for the whole hybrid deep neural network were used to predict the on-target knockout efficacy of a new sgRNA. In addition, in order to achieve rigorous evaluations of *DeepCRISPR*, both classification and regression models for on-target prediction were built for a comprehensive comparison.

#### Extending the model for sgRNA off-target site prediction by reusing the parent network

We also extended the hybrid neural network for sgRNA off-target profile prediction by reusing the pre-trained parent network (Fig. 1c, d). First, we treated a given sgRNA and its one possible off-target locus as a sample pair, and these sample pairs are taken as the off-target training samples. The sample pair was encoded in two parts, where one part represents the encoding of the given sgRNA and the other represents the encoding of its possible off-target locus (Fig. 1c; see the “sgRNA encoding with genome and epigenetic features” section). Such a two-part encoding presents an accurate and comprehensive representation of an off-target sample by considering the original sgRNA sequence and mismatched sequence as a whole. During training, each part of the sgRNA off-target sample was fitted into the pre-trained *DCDNN*-based network, i.e., the parent network, for feature representation learning. Next, the outputs of this parent network were combined together channel-wise for the following *CNN* classifier, similar to on-target site prediction (Fig. 1c). In our study, the complete hybrid neural network was trained based on the collected labeled sgRNA off-target datasets containing ~ 160,000 samples (see the “Off-target data sources” section). An efficient bootstrapping algorithm was integrated into the batch training of this hybrid network, alleviating the data imbalance issue in off-target site prediction (Fig. 5; see the “Integrating bootstrapping into batch training of deep neural networks to address the data imbalance issue” section). Similar to that of on-target site prediction, the training procedure not only learned the weights for the *CNN* network, but also tuned the weights of the parent network, resulting in two different “baby networks” for two parts of the sgRNA off-target sample. The final tuned weights for the two baby networks as well as the *CNN* network were used to predict the off-target profile of a given sgRNA. Similarly, in order to achieve rigorous evaluations of *DeepCRISPR*, both classification and regression models for off-target prediction were built for a comprehensive comparison.

### Comparison of *DeepCRISPR* with state-of-the-art sgRNA on-target efficacy prediction

To evaluate the ability of *DeepCRISPR* in sgRNA on-target efficacy prediction, we first curated comprehensive sgRNA on-target knockout efficacy benchmark data for humans, including four different cell types: i.e., *hct116* [36], *hek293t* [2], *hela* [36], and *hl60* [37]. Note that such datasets were also used by Haeussler et al. [16] for the benchmark study. The whole dataset includes ~ 15,000 sgRNAs with experimentally validated known knockout efficacies from 1071 genes. In our study, we formulated *DeepCRISPR* either in a classification schema or in a regression schema for a comprehensive and rigorous comparison. For the classification model, the known knockout efficacy was labeled in a binary way (see the “On-target data sources” section). For the regression model, the known knockout efficacy was integrated and labeled in a numerical way (see the “On-target data sources” section). Then, eight different testing scenarios were carefully designed for comprehensive and objective comparisons of *DeepCRISPR* with state-of-the-art tools. Through such comparisons, we provide solid evidence that (1) the deep learning models (without unsupervised pre-training) are superior to shallow learning models; (2) the unsupervised pre-training strategy boosts model performance; (3) the data augmentation further improves model performance and model robustness; (4) *DeepCRISPR* generalized generally well in new cell types for sgRNA on-target knockout efficacy prediction; (5) *DeepCRISPR* efficiently learns the high-level feature representation by avoiding manual feature engineering for sgRNA design, indicated by the apple-to-apple comparisons with the retrained *sgRNA designer* (the gradient-boost-based classification or regression models) with the same training data, while with different features; (6) *DeepCRISPR* is robust with superior performance for both classification and regression models.

#### Testing scenario 1—classification schema

**Fig. 2:**
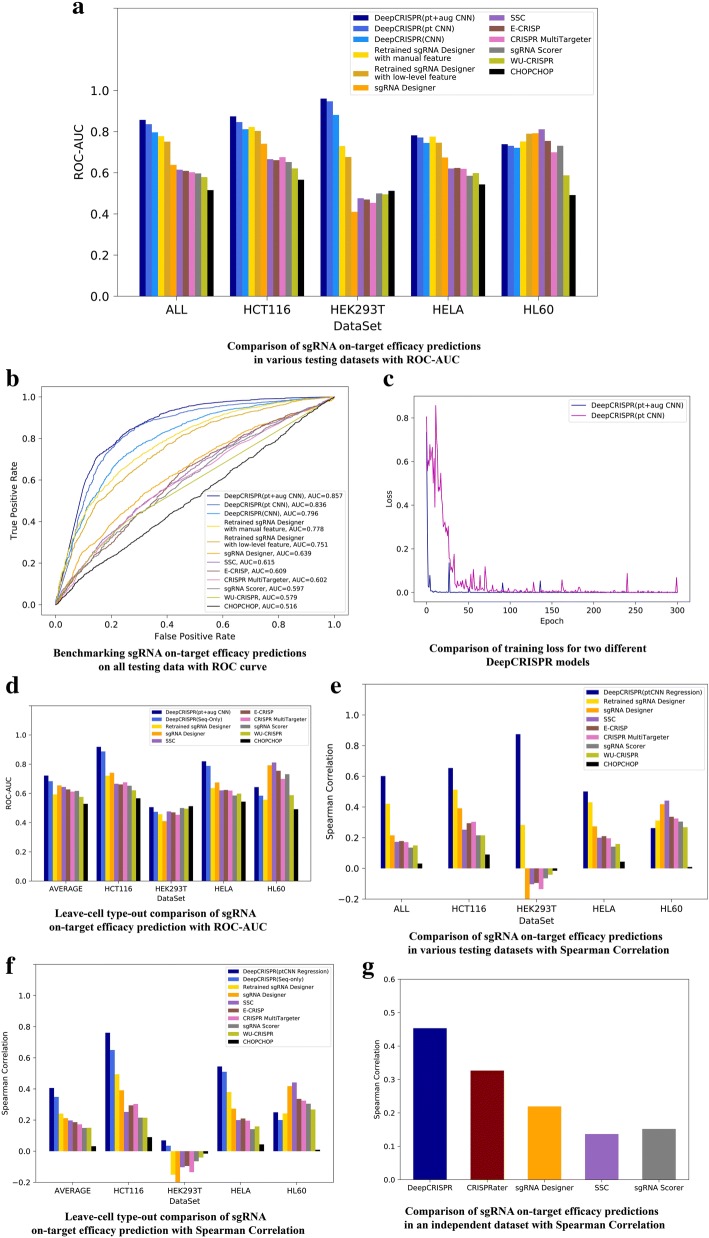
Evaluation of *DeepCRISPR* for on-target efficacy prediction. **a**, **b** Comparison of sgRNA on-target efficacy predictions in a classification schema for various datasets, i.e., *hct116* cell line, *hek293t* cell line, *hela* cell line, *hl60* cell line, and the overall testing dataset. **c** Comparison of training loss for two different *DeepCRISPR* classification models. **d** Leave cell type out comparison of sgRNA on-target efficacy prediction in a classification schema. **e** Comparison of sgRNA on-target efficacy predictions in a regression schema for various datasets, i.e., *hct116* cell line, *hek293t* cell line, *hela* cell line, *hl60* cell line, and the overall testing dataset. **f** Leave cell type out comparison of sgRNA on-target efficacy prediction in a regression schema. **g** Comparison of sgRNA on-target efficacy predictions in an independent dataset with Spearman correlation

In this test, for the original ~ 15,000 sgRNAs with known knockout efficacies from four cell types, 20% of the data from each cell type were stratified by data labels and used as independent testing sets. The remaining 80% of the data from each cell type were combined together for model training and parameter tuning during the cross-validation process. The deep *CNN*-based classification model without unsupervised pre-training and data augmentation (denoted as “CNN”) was trained and tested on the independent test data for the four cell lines, respectively, and compared with eight state-of-the-art tools that were trained with human cell line data, including *sgRNA Designer* [2], *SSC* [5], *CHOPCHOP* [10], *CRISPR MultiTargeter* [38], *E-CRISP* [9], *sgRNA Scorer* [12], *Cas-Designer* [39], and *WU-CRISPR* [40] (Fig. 2a, b)*.* These tools cover all the available methods designed for human sgRNA efficacy prediction, either by a learning model or a hypothesis-based scoring function (see Additional file 1 for a comprehensive list of current tools and the reasons we selected these tools for comparison). The comparison was evaluated using the values from the area under the receiver operating characteristic (*ROC*) curve (*AUC*) [41]. The comparison indicated that, on average, *DeepCRISPR* reached an overall *ROC-AUC* of 0.796, outperforming all eight methods with a maximum improvement of ~ 113% over *sgRNA Designer* [2] (with 0.5 *ROC-AUC* as the baseline), which is the next highest performing tool (Fig. 2a, b, Additional file 2).

#### Testing scenario 2—classification schema

In this test, we further built our model with unsupervised pre-training on ~ 0.68 billion unlabeled sgRNAs (denoted as “pt CNN”). The same training and testing data were used as for testing scenario 1. The overlapping sgRNAs between the training and testing data were removed. The pre-trained *CNN* reached an overall *ROC-AUC* of 0.836 with a 142% improvement over *sgRNA designer* (with 0.5 *ROC-AUC* as the baseline; Fig. 2a, b; Additional file 2).

#### Testing scenario 3—classification schema

We further built our final *DeepCRISPR* model with pre-training-based *CNN* plus data augmentation (denoted as “pt + aug CNN”). The training data were augmented while the testing data were identical to those of testing scenarios 1 and 2. The overlapping sgRNAs between the training and testing data were removed. For this case, *DeepCRISPR* reached an overall *ROC-AUC* of 0.857, with a 157% improvement over *sgRNA designer* (with 0.5 *ROC-AUC* as the baseline; Fig. 2a, b; Additional file 2). It can be seen that the improvement in performance was relatively small compared with testing scenario 2, while we found that the loss function during training converged fast and became very robust compared with that of testing scenario 2 (Fig. 2c). This indicates that increasing the label data amount can help to make the model robust and converge fast during the training.

#### Testing scenario 4—classification schema

In this scenario, we further tested the generalization ability of *DeepCRISPR* in new cell types. For the original ~ 15,000 sgRNAs with known knockout efficacies from four cell types, 20% of the data from each cell type were stratified by data labels and used as independent testing sets. The remaining 80% of the data from different cell types were augmented as the training data, identical to that of testing scenario 3. Then our model was trained in a fourfold “leave one cell type out” way, each time using the training data combined from three cell types and testing on the leave one cell type out independent dataset. The overlapping sgRNAs between training and testing data were removed. This testing scenario investigates the generalization ability of *DeepCRISPR* on new cell types (Fig. 2d, Additional file 2). For this case, the performance of *DeepCRISPR* on four cell types reached an average *ROC-AUC* of 0.722, outperforming the second best method, *sgRNA designer*. It can be seen that for *hct116* and *hela* cell types, the performance of *DeepCRISPR* was pretty good. For the *hek293t* cell type, all the test tools (including *DeepCRISPR*) performed poorly, mainly due to this cell type containing the majority of samples. Therefore, training models without such cell type data are inefficient with insufficient training data. Furthermore, in order to investigate whether the cell type-specific features, i.e., the cell-specific epigenetic features, really add to the performance of *DeepCRISPR*, we retrained *DeepCRISPR* without epigenetic features (i.e., the Seq-only *DeepCRISPR* model in Fig. 2d) for performance comparison. It can be seen in this case that the performance of the Seq-only *DeepCRISPR* model dropped slightly compared to the original one, indicating that (1) the cell-specific epigenetic features do add to the performance of *DeepCRISPR* and (2) the contribution to the prediction performance of adding cell-specific epigenetic features seems less than that of increasing the training data amount, as can be seen for the HEK293T cell type. *DeepCRISPR* performed moderately in the HL60 cell type. Since most other tools (including *sgRNA designer*, *SSC*, etc.) were trained based on HL60 data, their performance was generally better than *DeepCRISPR* in this specific cell type. As a summary, we conclude that *DeepCRISPR* performed generally well in new cell types for sgRNA on-target knockout efficacy prediction.

#### Testing scenario 5—classification schema

In this test, we provide a more rigorous and solid apples-to-apples comparison of *DeepCRISPR* with *sgRNA designer*, the next best tool during our former tests.

Firstly, we rigorously kept an identical comparison environment for *DeepCRISPR* and *sgRNA designer* with the same training and testing data. For this case, we retrained *sgRNA Designer* (https://github.com/MicrosoftResearch/Azimuth, a gradient boost classification-based shallow model) with the same augmented labeled dataset as *DeepCRISPR* used in testing scenario 3, and also kept the testing data identical. Then the following two different feature representations were performed: (1) we encoded the sgRNA with our one-hot feature representation (denoted as “retrained *sgRNA designer* with low-level feature”). This model achieved an overall *ROC-AUC* of 0.751 (Fig. 2a, b, Additional file 2); (2) we encoded the sgRNA with the original manually engineered features adopted by *sgRNA designer* (denoted as “retrained *sgRNA designer* with manual feature”). This model achieved an overall *ROC-AUC* of 0.778 (Fig. 2a, b, Additional file 2). Compared with these two different feature representations, it is indicated that the low-level feature encoding is not suitable for shallow models; therefore, the retrained *sgRNA designer* achieved better performance with manual domain-based feature engineering and feature encoding. Nevertheless, these results further indicate that the deep learning model can efficiently learn the high-level feature representation from low-level features and compete with the shallow models by avoiding manual feature engineering for sgRNA design.

Secondly, we also performed a leave one cell type out comparison of *DeepCRISPR* with retrained *sgRNA designer* using our one-hot feature representation. The test was performed on the same training and testing data as those of testing scenario 4 (Fig. 2d, Additional file 2). It can be seen that, on average, *DeepCRISPR* still outperformed retrained *sgRNA designer*, indicating its on-target prediction superiority compared to other methods.

#### Testing scenario 6—regression schema

In this test, we further trained *DeepCRISPR* in a regression schema with the original numerical sgRNA knockout efficacies. The data from different experiments were integrated in an elegant way as demonstrated in the “On-target data sources” section. The performance was evaluated with *Spearman correlation* as adapted in former studies [42]. The whole comparison was performed in a similar way as in testing scenarios 3 and 5, except that the model was trained in a regression way. Also the *sgRNA designer* was retrained in a regression way with the same training and testing data. It can be seen that in this case *DeepCRISPR* still outperformed the other methods as evaluated by Spearman correlation (Fig. 2e, Additional file 2).

#### Testing scenario 7—regression schema

We further tested the regression-based *DeepCRISPR* in a leave one cell type out way to investigate its generalization ability in new cell types, similar to test scenario 4. For this case *DeepCRISPR* achieved similar performance to those in a classification schema and outperformed the other methods as evaluated by Spearman correlation (Fig. 2f, Additional file 2).

#### Testing scenario 8—regression schema on an independent dataset

Since all the former tests (scenarios 1–7) were performed on the four cell types (*hct116*, *hek293t*, *hela*, and *hl60*) by separating the data for training and testing, in this case we applied an additional dataset which was totally independent of our former tests to investigate the on-target prediction performance of *DeepCRISPR*. This dataset, reported recently by utilizing fluorescent reporter knock-out assays with verification at selected endogenous loci for sgRNA knockout efficacy measurement, contains a total of 425 sgRNAs for HEL cells [43]. Both the cell type and data distribution are different to our former tests, and the sgRNAs do not overlap the former datasets. Therefore, it can serve as an ideal independent testing dataset to investigate the generalization ability of *DeepCRISPR*. In this test, we retrained *DeepCRISPR* with only sequence-level features on the original four cell type datasets, since the epigenetic features of the tested HEL cell type are not available in ENCODE. The retrained *DeepCRISPR* model was tested on this HEL cell data and compared with *sgRNA designer*, *SSC*, *sgRNA scorer*, and *CRISPRator*. Surprisingly, *DeepCRISPR* not only significantly outperformed *sgRNA designer*, the current state-of-the-art on-target prediction tool, with a nearly twofold improvement measured with Spearmen correlation, but also outperformed *CRISPRator*, which is designed specifically for this HEL cell dataset [43] (Fig. 2g, Additional file 3). This independent test further indicates that *DeepCRISPR* has good generalization ability for unseen data, even without the contribution of cell type-specific features.

In summary, for both the classification and regression models, *DeepCRISPR* generally outperformed alternatives for on-target prediction as measured by *ROC-AUC* and Spearman correlation. Also, it has a good cell type generalization ability. In addition, it can be seen that the amount of training data influences model performance, and the potential of deep learning models can be boosted with larger amounts of training data.

### Evaluation of *DeepCRISPR* for whole-genome sgRNA off-target profile prediction

We next evaluated the ability of *DeepCRISPR* to predict off-target sites. For this purpose, we curated the human sgRNA whole-genome off-target profile data detected by *GUIDE-seq,Digenome-seq*, *BLESS*, *HTGTS*, and *IDL*V. These data include 30 sgRNAs from two different cell types: the HEK 293 cell line and its derivatives (18 sgRNAs) [6, 22, 24–27], and K562 t (12 sgRNAs) [44], accounting for ~ 160,000 possible loci with a maximum of six nucleotide mismatches (see the “Off-target data sources” section)*.*We also formulated *DeepCRISPR* in a classification and a regression schema for a comprehensive and rigorous comparison. For the classification model, the off-target sites are labeled as “1” and the others are labeled as “0” (see the “Off-target data sources” section). For the regression model, the off-target sites are labeled with the targeting efficacies measured with indel frequency detected by different assays (see the “Off-target data sources” section). Then three different testing scenarios were designed for off-target profile prediction evaluation.

#### Testing scenario 1

**Fig. 3:**
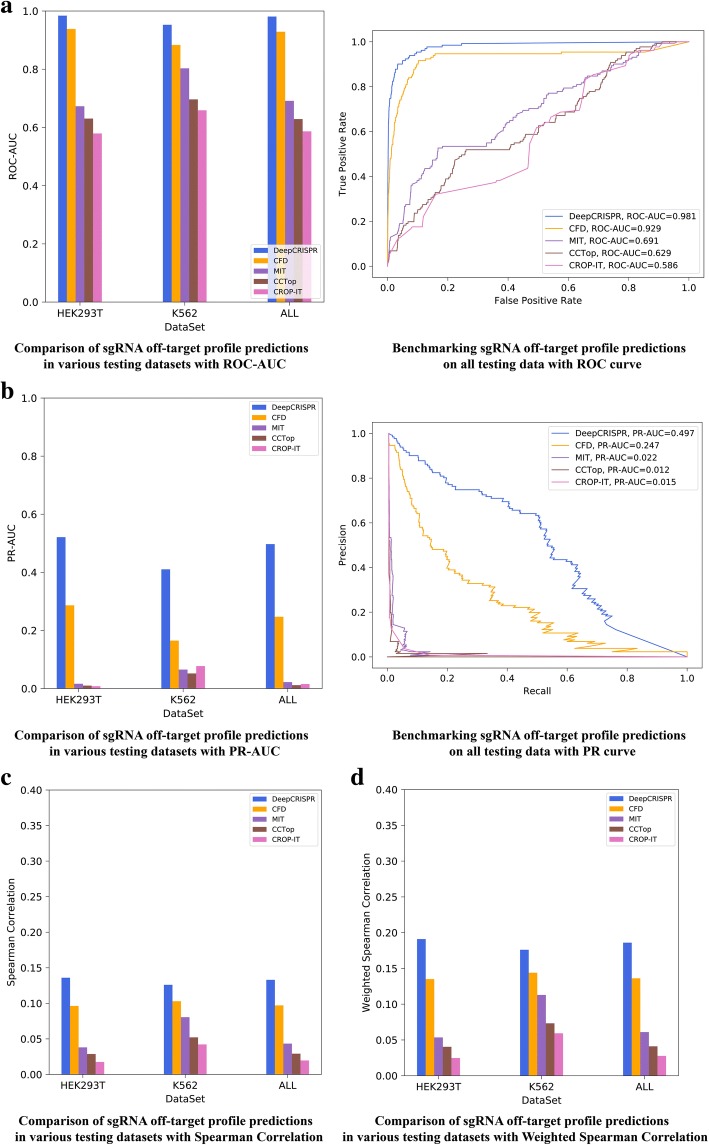
Evaluation of *DeepCRISPR* for off-target profile prediction. **a** Comparison of sgRNA off-target profile predictions in a classification schema for various datasets, i.e., *293*-related cell types and *K562* cell line with a maximum of six mismatches. The performance was evaluated with *ROC-AUC*. **b** Comparison of sgRNA off-target profile predictions in a classification schema for various datasets, i.e., *293*-related cell types and *K562* cell line with a maximum of six mismatches. The performance was evaluated with *PR-AUC*. **c** Comparison of sgRNA off-target profile predictions in a regression schema for various datasets, i.e., *293*-related cell types and *K562* cell line with a maximum of six mismatches. The performance was evaluated with Spearman correlation. **d** Comparison of sgRNA off-target profile predictions in a regression schema for various datasets, i.e., *293*-related cell types and *K562* cell line with a maximum of six mismatches. The performance was evaluated with weighted Spearman correlation

We withheld 20% of the data for each cell type as an independent testing set. The remaining 80% of data were combined together to train our model and tune the parameters during the cross-validation process. Because the whole dataset was highly unbalanced with ~ 700 true off-target sites, an efficient bootstrapping sampling algorithm was adapted in the training procedure to alleviate the data imbalance (see the “Integrating bootstrapping into batch training of deep neural networks to address the data imbalance issue” section). As a result, our final trained off-target site prediction model was tested on the independent datasets for each of the two cell lines and compared with four of the current state-of-the-art off-target site prediction tools, *CFD score* [2], *MIT score* [6], *CROP-IT* [45], and *CCTop* [46]*.*These tools were designed for human sgRNA off-target site prediction using various empirically defined off-target scores. Since the whole dataset is unbalanced, the comparison was evaluated using *AUC* values from the *ROC* and precision-recall curve for the classification model, and Spearman correlation and weighted Spearman correlation [42] for the regression model. For a maximum of six nucleotide mismatches, the testing results indicated that *DeepCRISPR* outperformed all four methods in the two cell types (Fig. 3a-c, Additional file 2). Overall, *DeepCRISPR* achieved an *ROC-AUC* of 0.981(Fig. 3a), *PR-AUC* of 0.497(Fig. 3b), Spearman correlation of 0.133 (Fig. 3c) and weighted Spearman correlation of 0.186 (Fig. 3c), outperforming the second-best method, i.e., the *CFD* score [2] (Fig. 3a-c).

It is worth noting that the improvement in off-target prediction with *DeepCRISPR* is a relatively small margin when compared with the *CFD* score evaluated with *ROC-AUC* since the *CFD* score had already achieved high performance; nevertheless, such improvement is very important since near-zero off-targeting is the ultimate goal for all CRISPR-based gene therapies. Also, it should be noted that all the existing tools, including *DeepCRISPR*, tend to avoid missing true off-target cleavage sites by weighting higher on positive samples. This also makes sense for CRISPR-based gene therapy, as the penalty of missing a true off-target site is always higher than that of inducing a false positive in off-target site prediction. That is why we adopted the weighted Spearman correlation proposed by Listgarten et al. [42] to address such weight asymmetry issues. The weight for each off-target site is set proportional to its rank order according to the corresponding knock-out efficacy measured by indel frequency. Nevertheless, such a weighting schema is actually a compromise for false positives. Therefore, reducing false positives purely from unweighted data is still required and is very challenging. For this case, it can be seen that *DeepCRISPR* greatly improved the *PR-AUC* value compared to the other methods, indicating that *DeepCRISPR* can dramatically reduce the false positives during off-target prediction..

#### Testing scenario 2

**Fig. 4:**
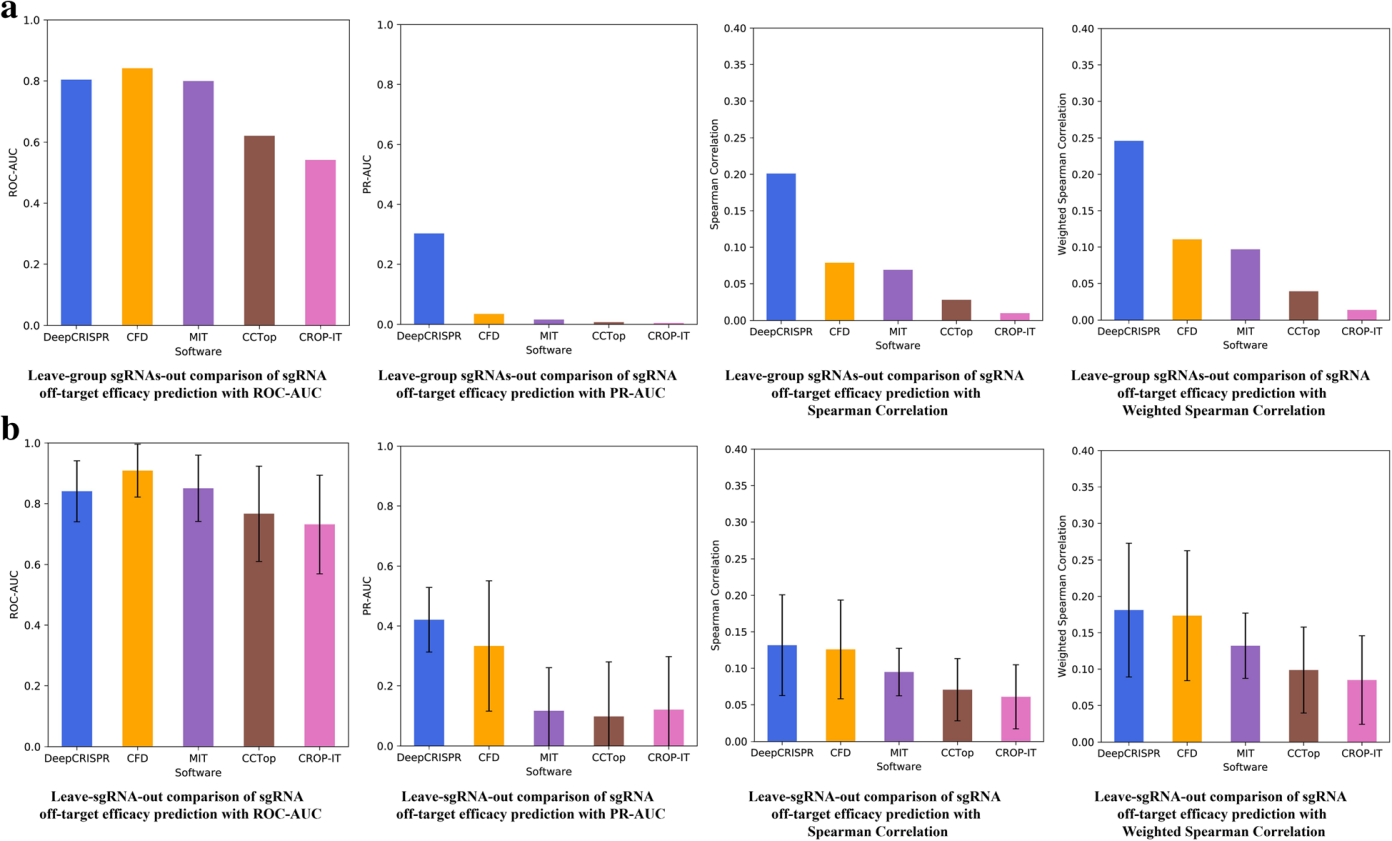
**a** Leave sgRNAs group out comparison of sgRNA off-target efficacy prediction with *ROC-AUC*, *PR-AUC*, Spearman correlation, and weighted Spearman correlation. **b** Leave sgRNA out comparison of sgRNA off-target efficacy prediction with *ROC-AUC*, *PR-AUC*, Spearman correlation, and weighted Spearman correlation. The error bars in Fig. 4b indicate the variances of the average performances in different tests

In this scenario, for all the 30 sgRNAs from two different cell types, we performed a “leave sgRNA group out” test, which is a more representative use case for off-target profile detection. Such a test randomly holds a group of sgRNAs out (in our case three sgRNAs were held out) as testing data, presenting an estimate of the predictive performance on a group of unseen sgRNAs (Fig. 4a). It ensures that the off-target guides for one sgRNA are either entirely in the test or training sets. In this case, for both classification and regression models, *DeepCRISPR* achieved an average *ROC-AUC* of 0.804, *PR-AUC* of 0.303, Spearman correlation of 0.201, and weighted Spearman correlation of 0.246 (Fig. 4a, Additional file 2). The *ROC-AUC* for *DeepCRISPR* is comparable to the result with *CFD score*, while other measurements, especially the *PR-AUC* (0.303), are significantly higher than with *CFD score* (0.034), indicating that *DeepCRISPR* can help to reduce false positives for unseen sgRNAs in off-target prediction.

#### Testing scenario 3

In this scenario, for all 30 sgRNAs, we performed 30-fold leave one sgRNA out testing, which is an extreme case of the leave sgRNA group out test as shown in testing scenario 2 (Fig. 4b). For both classification and regression models, *DeepCRISPR* achieved an average *ROC-AUC* of 0.841, *PR-AUC* of 0.421, Spearman correlation of 0.132, and *weighted Spearman correlation* of 0.181 (Fig. 4b, Additional file 2). In this case, the *ROC-AUC* of *DeepCRISPR* is comparable to the result from *CFD score*, while other measurements, especially *PR-AUC* (0.421), are higher than with *CFD score* (0.333).

In summary, for both classification and regression models, *DeepCRISPR* generally outperformed *CFD* score, especially with improved performance to reduce false positives in the highly imbalanced off-target prediction. One thing to note is that the classification model is more suitable for off-target prediction compared to the regression model since in this case we only care about distinguishing off-target sites among others rather than predicting their binding affinities. In addition, the regression model is more sensitive and thus requires more data to train it. The current version of *DeepCRISPR* has only been trained on limited samples as a prototype study. We are expecting to boost *DeepCRISPR* with more training samples, taking full advantage of deep models compared to shallow models.

## Automating feature identification in a learning schema

We intended to automate the whole feature identification procedure purely based on the available training data and the learning model. It should be noted that feature identification and visualization based on shallow learning models have been extensively addressed, and numerous works have been presented to select features for in silico sgRNA design [1, 2, 5]. However, feature identification and interpretation for deep learning models is non-trivial and worthy of exploration. In our study, we present a computational method to derive the feature saliency map for efficient sgRNA design with optimization [47] based on the trained deep learning model. We allow the trained deep neural network model to tell us what the efficient sgRNAs look like compared to inefficient sgRNAs (see the “Feature identification by deriving a class-specific feature saliency map” section).

**Fig. 5:**
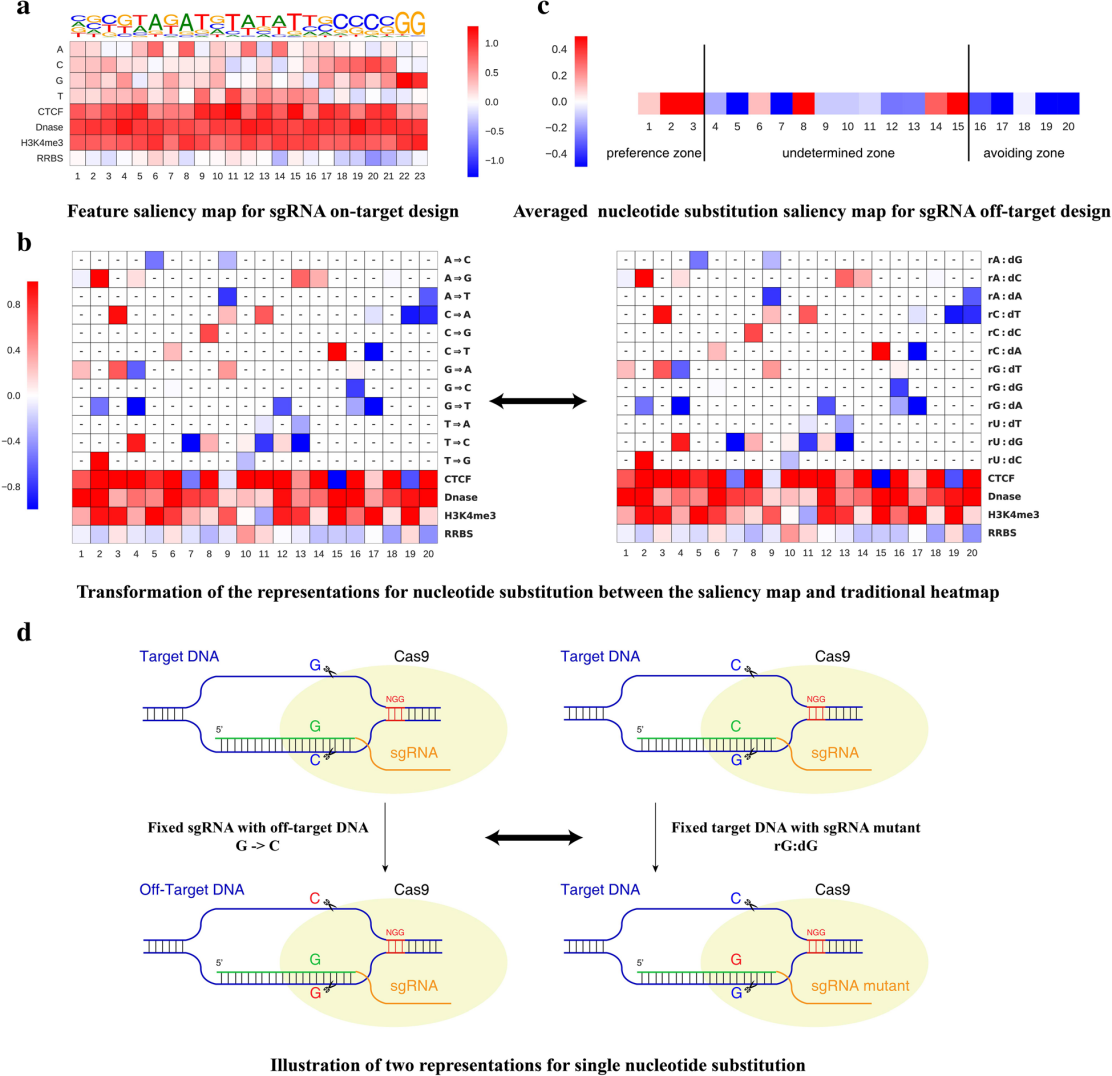
Automatic learning sequence and epigenetic features affecting sgRNA on-target and off-target design. **a** Feature saliency map for sgRNA on-target design. **b** Transformation of the representations of nucleotide substitution between the saliency map and traditional heatmap. *Hyphens* in both maps indicate that the impact of the nucleotide substitution at this position is not statistically significant by a Fisher’s exact test. **c** Averaged nucleotide substitution saliency map for sgRNA off-target design. Three nucleotide substitution zones are clearly identified. **d** Two representations for single nucleotide substitution (see the “Feature identification by deriving a class-specific feature saliency map” section

We first generated the feature saliency map for sgRNA on-target site prediction based on the existing training data, as shown in Fig. 5a. We obtained a feature saliency map consistent with previous findings: (1) it has the same preferences in the variable nucleotides of the PAM NGG for high efficacy sgRNA, where cytosine is favored and thymine is disfavored. This is consistent with existing in vitro and in vivo studies [1, 2]. (2) Thymines are disfavored at the four positions closest to the PAM, consistent with the fact that multiple uracils in the spacer lead to low sgRNA expression [1]. (3) Position 18 has a consistent preference for cytosine, which is the DNA cleavage site of CRISPR system [5, 37]. (4) It has a general preference for an open chromatin structure, as indicated by the feature saliency map of CTCF, DNase, and H3K4me3. (5) It has a relatively avoidance of DNA methylation for high sgRNA efficacy, as shown by the RRBS assay. This is consistent with a recent study exploring various features for CRISPR off-targets [28]. In summary, nucleotide preferences at specific positions coupled with an open chromatin structure are preferred for optimized sgRNA on-target design.

For off-target site prediction, a detailed feature saliency map for 16 possible nucleotide substitutions across 20 positions is presented, by filtering those points without statistical significant using a Fisher statistical test (Fig. 5b; see the “Feature identification by deriving a class-specific feature saliency map” section). We also generated an averaged nucleotide substitution rate map to indicate their effect on the occurrence of off-target cleavage (Fig. 5c; see “Feature identification by deriving a class-specific feature saliency map” section). We divided this feature map into three nucleotide substitution zones, i.e., *off-target preference zone* (positions 1–3), *undetermined zone* (positions 4–15), and *off-target avoiding zone* (positions 16–20). Although this map was obtained from limited samples, we observed that the nucleotide mutations occurring near the PAM are prone to avoid off-target sites in a position and nucleotide identity-dependent manner. This is consistent with previous findings that changing the nucleotides far from the PAM usually has little effect on sgRNA efficacy [2, 6]. Previously, two different groups performed extensive in vitro tests on human EMX1 and CD33 genes to generate different guide RNAs containing possible single-nucleotide substitutions for off-target studies. Their study indicated that SpCas9 tolerates single-base mismatches in the PAM distal region to a greater extent than in the PAM proximal region [2, 6].

Specifically, the learned feature map (Additional file 4) gave results consistent with previous studies and new findings. *DeepCRISPR* identified a preference for purine:purine mismatches to avoid off-target sites with statistical significance, including the substitution G->C(corresponding to rG:dG in a traditional heatmap, as previously reported [2]) and substitution G->T (corresponding to rG:dA in a traditional heatmap) at position 16. Kinetic studies of CRISPR dynamics in living cells also proved that the purine and purine mismatches at position 16 radically reduce binding affinity and diminish cleavage activities [48]. Besides these consistent findings, our feature saliency map identified five nucleotide substitutions preferring off-targets in the *off-target preference zone* and eight nucleotide substitutions avoiding off-targets in the *off-target avoiding zone* (Fig. 5b, c), including the two nucleotide substitutions G->C and G->T at position 16 [48]. Future validation of these nucleotide substitutions based on large-scale off-target data is expected and the identified factors will become more accurate with more off-target data in the future.

The interpretation of Fig. 5b is different from that of previous studies, which is explained in the "Feature identification by deriving a class-specific feature saliency map" section.

## Discussion and conclusions

Here we present *DeepCRISPR*, an efficient and extendable computational model for simultaneous prediction of CRISPR sgRNA on-target knockout efficacy and whole genome off-target profiles. *DeepCRISPR* surpassed the state-of-the-art tools across a variety of human datasets with solid evaluation metrics. Importantly, our results indicate that leveraging genome-wide unlabeled sgRNA sequences as well as a deep learning model helps to efficiently learn sgRNA representations and boost the prediction performance. In addition, *DeepCRISPR* automates the feature identification for sgRNA design in a data-driven manner, facilitating interpretation and optimized CRISPR on-target and off-target design.

A number of future improvements are expected: (1) currently we have designed only a relatively simple and concise unsupervised pre-training and *CNN*-based deep neural network model. Various complex and modern deep learning architectures await exploration in the future; these models are expected to improve our current prediction performance. Nevertheless, the main goal of this study is to provide a prototype and applying more sophisticated deep learning models is encouraged in the future. (2) Manual design of proper sgRNA features will definitely help to boost the sgRNA efficacy prediction, although this is not our main goal here. In our study, we use only the low-level one-hot feature encoding sgRNA representations, and we want to show the prediction ability of deep learning models rather than the contribution of feature engineering. Future directions can combine feature representation learning and feature engineering for improved prediction performance. (3) On-target training data may contain noise. The published sgRNA screens may not exactly measure the sgRNA knock-out efficacy, which may be taken as a confounding effect waiting to be further explored. Nevertheless, several efforts have been made to improve the knockout measurements [12, 43]. Our independent validation of the refined sgRNA on-target dataset utilizing fluorescent reporter knock-out assays [43] also indicated that *DeepCRISPR* surpassed other current methods (Fig. 2g). Further improved prediction model building with more accurate knockout labels is expected. (4) For off-target prediction, different off-target assays may have different sensitivities. The assays likely do not capture the full space of off-target cleavage sites. Compared to on-target prediction, this might be inherently difficult since the current off-target raw datasets are not solid. Furthermore, the amount of off-target raw data might be less sufficient than the datasets for on-target prediction. In our study we integrated data from different off-target detection platforms and such a data collection schema is currently well-accepted [1, 5].

The main point of our current study is to present *DeepCRISPR* as the first prototype model that demonstrate the utility of deep learning for optimized sgRNA design. Nevertheless, better noise-free off-target data are waiting to be accumulated. Higher sensitive off-target detection techniques are expected to be developed. Our models can be trained based on these data for improved CRISPR off-target prediction in the future.

In addition, the amount of available sgRNA knockout data is relatively small, which provides a challenge for training a routine deep learning model. A common concern with deep models is that they can overfit the training data [31]. Therefore, besides the previous collection of genome-wide sgRNA sequences for unsupervised representation learning, we carefully designed a hybrid deep network incorporating several other techniques: (1) an efficient data augmentation technique to increase the training sample size; (2) fine-tuning-based improvement of the model’s generalization ability; and (3) efficient *batch normalization* [31, 32] techniques to avoid overfitting. By using these techniques, we surpassed the current state-of-the-art tools, both in on-target and off-target site prediction. On the other hand, we are anticipating a rapid increase in the amount of CRISPR genome editing data due to the booming popularity of this new technique. For this reason, the performance of *DeepCRISPR* is expected to be enhanced with the availability of more training data, taking advantage of this powerful deep learning framework. This is a superior feature of *DeepCRISPR* compared with shallow learning models [32]. We believe that future insights from the deep learning community as well as the data accumulation in the genome editing community will lead to enhancements of *DeepCRISPR* and CRISPR-based gene editing analysis generally.

## Methods

### Data collection and processing

#### On-target data sources

The initial on-target dataset contains seed sgRNAs with experimentally validated known knockout efficacy, comprising ~ 15,000 sgRNAs containing 1071 genes from four different cell lines (*hct116* [36], *hek293t* [2], *hela* [36], and *hl60* [37]) with redundancy removed. The sgRNA knockout efficacy measurements were restricted to experimental assays, where the efficacy is defined as the log-fold change in measured knockout efficacy. We excluded any other readouts of knockout measurements without in vivo or in vitro experimental validation, such as sequence-based readouts of the frameshift ratio, as their correlations with the true knockout efficiency are unclear [15]. For classification models, we converted sgRNA efficacy to a binary value using a log-fold change of 1 as the cutoff. Such label categorization was also applied previously [5]. For regression models, we adopted a collaborative filtering-based data normalization method, borrowing the idea from the user-item recommendation system [49, 50]. Specifically, a matrix *Y* is formulated where each row represents one of the experiments and each column represents one sgRNA. *y*_*ij*_ indicates the *j*th sgRNA knock-out efficacy in the *i*th experiment. First, three types of mean value are calculated, namely, mean values for each row, mean values for each column, and the mean value of the whole matrix. Then the normalized on-target efficacy values *y*_*norm*_ for *y*_*ij*_ are obtained by subtraction of the original on-target efficacy values and the weighted sum of mean values from the related row (*m*_*row*_) and column (*m*_*column*_) as well as the whole matrix (*m*_*all*_), which is shown in the following formula:

1 $$ {y}_{norm}={y}_{ij}-\left({m}_{row}+{m}_{column}+{m}_{all}\right)/3 $$

After calculating the normalized on-target efficacy values integrated from different experiments, a rank-based normalization [2] is performed to obtain the final numerical labels (Additional file 5).

#### Off-target data sources

The off-target profile dataset contained two different cell types: 293-related cell lines (18 sgRNAs) and K562 t (12 sgRNAs) [6, 22, 24–27, 44]. For all 30 sgRNAs, we obtained ~ 160,000 possible loci across the whole genome using *bowtie2* [51], with a maximum of six nucleotide mismatches*.* The whole dataset was highly unbalanced, and nearly 1/250 loci was identified as an off-target site (one mismatch, 4; two mismatches, 31; three mismatches, 121; four mismatches, 236; five mismatches, 174; six mismatches, 75) with various whole genome off-target detection techniques [22–27] (Additional files 6 and 7). For the classification model, the off-target sites are labeled as “1” and the others are labeled as “0”. For the regression model, the off-target sites are labeled and normalized with the targeting cleavage frequency (indel frequency) detected by different off-target detection assays.

#### sgRNA encoding with genome and epigenetic features

We formulated an efficient image-like coding scheme to encode a DNA region which contains both nucleotide sequence and epigenetic information. The aim was to consider epigenetic information by representing different DNA regions from different cell types with a unified feature space. These epigenetic features included CTCF binding information from the ChIP-Seq assay, H3K4me3 position information from the ChIP-Seq assay, chromatin-opening information from the DNase-Seq assay, and DNA methylation information from the RRBS assay, obtained from *ENCODE* [52]. In our study, we treated a DNA region as a one-row multi-channel picture. For a conventional colored picture, each pixel has three values, namely, red, green, and blue from three channels. For a DNA region “picture”, the nucleotide sequence is represented by four channels, i.e., the A-channel, C-channel, G-channel, and T-channel, and each epigenetic feature is considered as one channel. As a result, we obtained an eight-channel representation of each DNA region (Fig. 1a). Based on this encoding schema, the current version of *DeepCRISPR* supports genome-wide sgRNA efficacy prediction for 13 human cell lines (HEK293, MCF-7, K562, HL60, NB4, BE2C, Caco-2, GM06990, Hela, HCT116, LNCap, HepG2, and GM12878) with all required epigenetic information. It can be easily extended to other cell types if the required epigenetic information is provided.

#### Data augmentation for on-target dataset

The final on-target dataset was generated in a data augmentation manner like that used in image data processing. Considering that sgRNA with two mismatches in the first two positions from the 5′ end commonly has no effect on cleavage efficacy [1, 2], we extended the original seeds by changing each one into a new sgRNA with two mismatches in the PAM distal region. These newly generated sgRNAs with the same epigenetic profiles had identical efficacy labels to those of the seeds. This augmentation resulted into ~ 0.2 million non-redundant sgRNAs with biologically meaningful knockout efficacies for the training process (Additional file 8).

### Deep learning techniques

#### *DCDNN*-based autoencoder for representation learning

*DeepCRISPR* uses a *deep convolutionary denosing neural network* (*DCDNN*)-based autoencoder to learn the underlying representation of sgRNA regions, which contain both the DNA nucleotide sequence and epigenetic information. The autoencoder has an encoder and a decoder, which are in sequential order. Optimization of the autoencoder during training results in the underlying representation of the given sgRNAs [35]. In our study, the input of *DeepCRISPR* comprises all the sgRNA regional information of the whole human genome from 13 cell types collected from *ENCODE* [52], which accounts for over ~ 0.68 billion data samples (Fig. 1c). Detailed network descriptions are in Additional file 9.

#### *CNN* model with pre-training-based fine-tuning

Followed by the *DCDNN*-based autoencoder for sgRNA representation learning, *DeepCRISPR* adopted a fully *convolutional neural network* (CNN) model for predicting sgRNA efficacy (Fig. 1b-d). *DeepCRISPR* applies a *fine-tuning* strategy to train the model by utilizing an autoencoder-based model to be part of the classifier and then tune the whole classifier with labeled data. The general information learned by large-scale unlabeled data can be used to boost the prediction performance. Specifically, we used the encoder part of the *DCDNN*-based autoencoder as the pre-trained model. For on-target prediction, it contains the pre-trained *DCDNN*-based encoder as well as the *CNN* layers, and its input is the sgRNA regional information data. For off-target prediction, it contains two pre-trained *DCDNN*-based encoders, one merged layer, and *CNN* layers. The input for the off-target prediction comprises two parts: target sgRNA regional information data and off-target sgRNA regional information data. Each part is fed into a pre-trained *DCDNN*-based encoder and then their results are merged. The merged result is fed into the *CNN* layers to predict off-target sites (Fig. 1b-d). Detailed network descriptions are in Additional file 9.

#### Integrating bootstrapping into batch training of deep neural networks to address the data imbalance issue

**Fig. 6:**
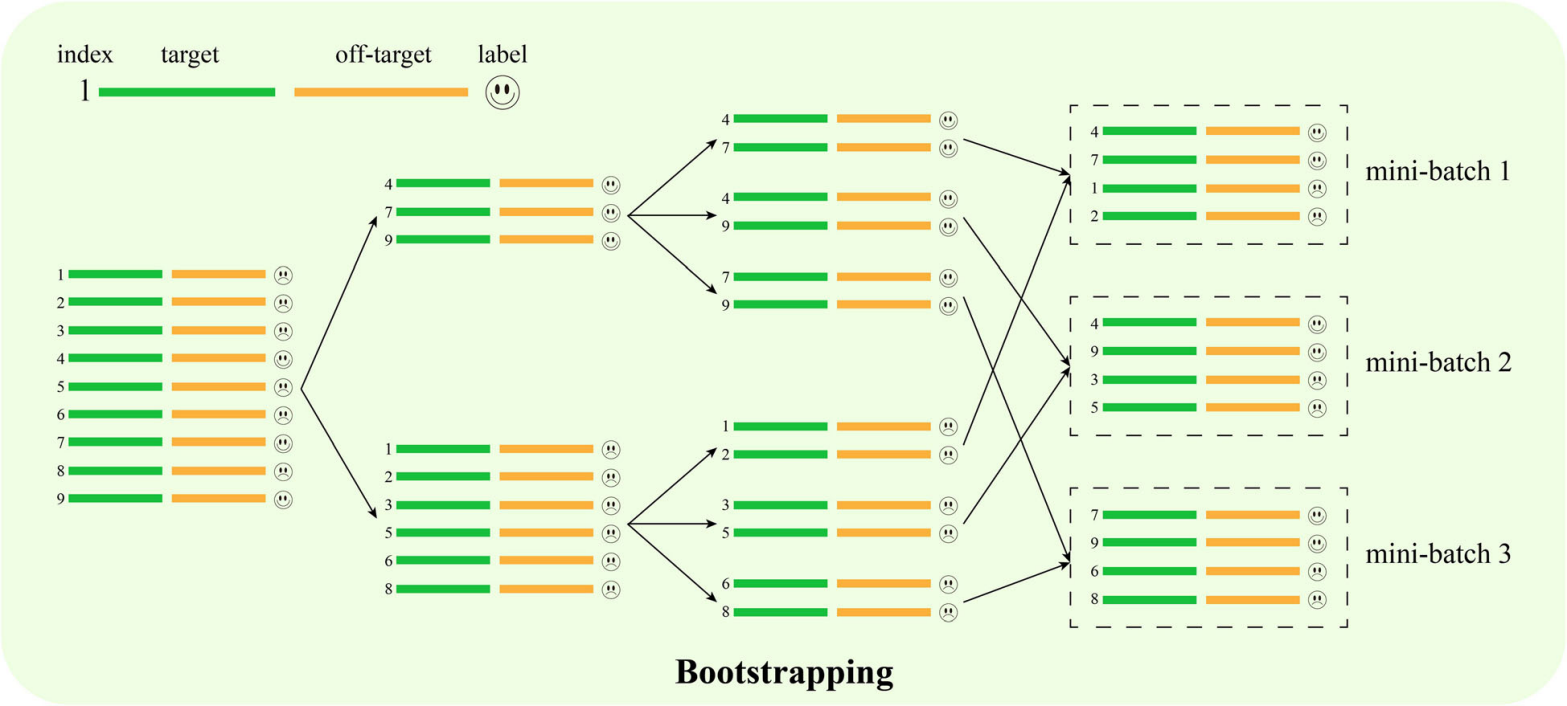
Bootstrapping sampling for sgRNA off-target site prediction. The bootstrapping sampling is performed from the minor samples to obtain the same amount of samples as major samples in each mini-batch

During the training for off-target site prediction, the data were highly unbalanced, i.e., the ratio of known off-target sites and negative off-target sites was approximately 1:250. Highly unbalanced data will probably make the gradient update unstable and eventually cause the model training to fail. To deal with this issue, *DeepCRISPR* applied a bootstrapping method to the mini-batches during the training process [32]. A similar idea has also been applied in related shallow learning models [28]. The basic idea of this method is to perform bootstrapping sampling from the minor samples and obtain the same amount of samples as major samples at the same time in mini-batches. This strategy ensures that the mini-batches have the same amount of positive samples and negative samples, thus avoiding gradient update instability and substantially boosting the prediction performance (Fig. 6).

#### Feature identification by deriving a class-specific feature saliency map

We generated positive class-specific saliency maps for on-target and off-target models (Fig. 4).Given the learned model *DeepCRISPR* and a class of interest (the class of efficient sgRNAs), feature identification is achieved by numerically generating a “synthetic sgRNA”, which is representative of the class in terms of the *DeepCRISPR* scoring model. Formally, let *S*_*c*_(*g*) be the score of the class *c*, computed by *DeepCRISPR* for a guide RNA *g*; we would like to find an optimized sgRNA, such that the score *S*_*c*_ is high:

2 $$ {argmax}_g{S}_c(g) $$

A locally optimal *g* can be found by a gradient ascent method with eq. (1). The basic idea is that we just let the model learn to determine the dominant features based on the training samples related to a specific class. It maximizes the model output with respect to input and the calculated input reveals the optimized result [47]. For the on-target model, the trained deep neural network model just tells us what the efficient sgRNAs look like compared to inefficient sgRNAs. For the off-target model, the trained deep neural network model tells us what a pair sample, i.e., <a given sgRNA, off-target> looks like. This is particularly helpful for us to derive the single nucleotide substitution saliency map (Fig. 5b). To better understand the off-target site prediction model, we integrated the saliency maps into one substation level map (Fig. 5c). Because the number of target sgRNAs for the off-target model is relatively small, we also performed a Fisher’s exact test to test the significance of the impact on off-target sites for every nucleotide substitution at different positions [53].

The interpretation of Fig. 5b is presented in the following: (1) traditional experimental studies often fix the DNA locus and generate different sgRNA mutants with single nucleotide substitutions to investigate their effect on cleavage efficacy. In our study, taking advantage of the two-channel encoding for off-target site prediction mentioned above, the model for off-target interpretation is learned as a whole from the training data. It considers the original sgRNA simultaneously with its corresponding detected off-target sites with nucleotide substitutions occurring at different positions. Thus, we can directly infer the impact of nucleotide substitution on off-target prediction. The epigenetic information should also be considered together with nucleotide substitution to investigate the effect on cleavage efficacy. (2) A transformation is carried out to compare our saliency map with the traditional heatmap for feature identification, as explained in Fig. 5d. The traditional experimental method uses a fixed DNA locus and investigates the binding cleavage efficacy for different sgRNAs with a nucleotide substitution at this locus [2, 6]. Our data-driven model, on the other hand, fixes the given sgRNA by generating different off-target sites detected by whole-genome profiling. The cleavage efficacy for a given fixed sgRNA on a different off-target locus is explored. For this reason, rG:dG as presented in a traditional heatmap can be referred to as the G->C substitution presented in our salient map (Fig. 5d). (3) Our data-driven procedure relies on existing training data. If the training data do not cover sufficient substitution samples in a specific position, the model will generally filter out such features.

#### Visualization of the off-target profile using *Circos* plot

We built an integrated web tool, *DeepCRISPR*, to unify human sgRNA on-target knockout efficacy prediction and off-target profile prediction in one framework. The *DeepCRISPR* platform has the following functions: (1) for a given sgRNA, *DeepCRISPR* accurately predicts its possible knockout efficacy at the corresponding DNA loci using a classification model; (2) for a given sgRNA, *DeepCRISPR* discovers its possible genome-wide off-targets and predicts their potential off-target cleavage efficacies using a classification model.

We also noticed that several tools have assigned a summary off-target score to describe the off-target level of a given sgRNA [2, 6, 42]. In our study, we also defined the “*anti-OT score*”, whose range is (0, 1]:

3 $$ S=\ln \left(1+{\mathrm{e}}^{\sum \left({OT}_i\right)}\right)/\mathit{\ln}2 $$

where *∑*() denotes the summation of the occurrence probability of a potential off-target candidate *OT*_*i*_ of a given sgRNA. A sgRNA having a higher a*nti-OT score* over another means that this sgRNA has a lower probability to have off-target cleavage across the whole genome.

**Fig. 7:**
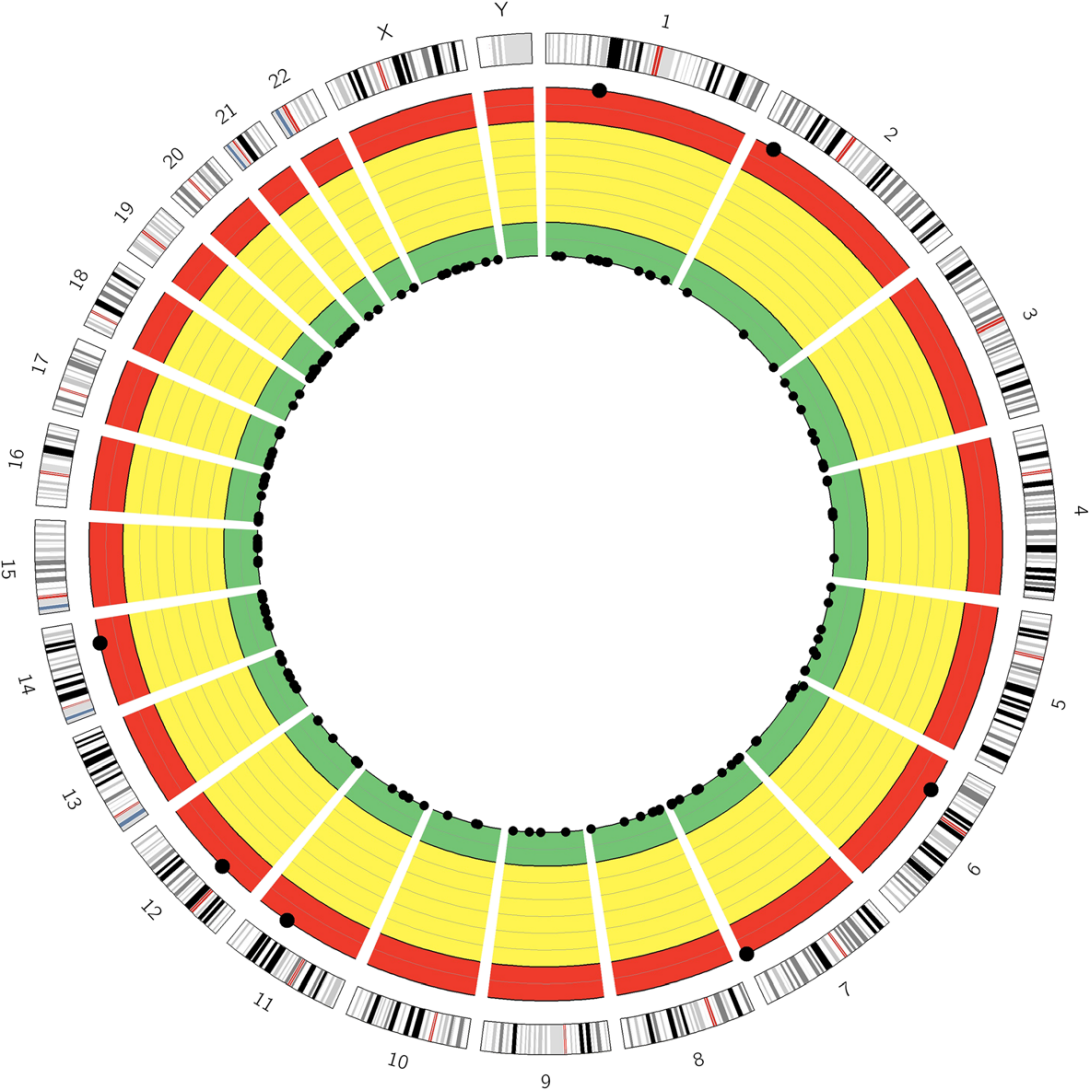
A *Circos* plot example visualizing the off-target profile of a given sgRNA (GCCTCTTTCCCACCCACCTTGGG) in the HEK293t cell type

In addition, we also provide a graphical demonstration by visualizing the overall genome-wide off-target profile of a given sgRNA with a *Circos* plot [54]. From the inner circle to the outer circle, different colors (green, yellow, and red) represent different off-target levels (mild, moderate, and severe) (Fig. 7). Such a visualization presents an intuitive way for users to access and evaluate the summary off-target profile of a given sgRNA.

## Additional files

Additional file 1: A comprehensive list of hypothesis-based and learning-based sgRNA on-target design tools and the selected candidates in our comparison study. (XLSX 14 kb)

Additional file 2: Detailed comparison results for sgRNA on-target efficacy prediction and off-target site prediction. (XLSX 16 kb)

Additional file 3: Comparison of sgRNA on-target efficacy predictions in an independent dataset with Spearman correlation. (XLSX 48 kb)

Additional file 4: Detailed learned weights for feature saliency map with filtering of those positions without statistical significant by Fisher’s exact test. (XLSX 6 kb)

Additional file 5: The datasets used for the study of sgRNA on-target efficacy prediction. (XLSX 959 kb)

Additional file 6: The dataset from the human 293-related cell type used for the study of sgRNA off-target profile prediction. (XLSX 49 kb)

Additional file 7: The dataset from the human K562 cell type used for the study of sgRNA off-target profile prediction. (XLSX 19 kb)

Additional file 8: Data augmentation for on-target dataset. (XLSX 2939 kb)

Additional file 9: Supplementary notes. (PDF 339 kb)

## Abbreviations

BLESS: Direct in situ breaks labeling sequencing
CNN: Convolutional neural network
HTGTS: High-throughput genome-wide translocation sequencing
IDLV: Integration-deficient lentiviral vector capture
OT: Off-target
PAM: Protospacer adjacent motif
sgRNA: Single-guide RNA
