# Supplementary material for: DeepCRISPR: optimized CRISPR guide RNA design by deep learning
Source: Genome Biol. 2018 Jun 26;19:80. doi: 10.1186/s13059-018-1459-4 (PMC6020378; doi:10.1186/s13059-018-1459-4)
Supplement: Supplementary file 9 — Supplementary notes. (PDF 339 kb) [file 13059_2018_1459_MOESM9_ESM.pdf]

Supplementary notes of the detailed model architecture

In our study, we established three major deep neural networks, including one *DCDNN*-based autoencoder for representation learning, two convolutional neural network with pre-training based fine-tuning for on-target prediction and off-target prediction respectively.

In our *DCDNN*-based autoencoder, we established 5 convolution layers as the encoder with batch normalization and normalized noise behind each convolution layer, and 5 deconvolution layers correspondingly as the decoder. The structure of the network is shown in Table S1. In comparison with common dense-layer-based denoising autoencoder, this DCDNN-based autoencoder highly utilized batch normalization and injected normalized noise into every layer in the encoder part which could make representation learning better and robust. Also, using convolution layer instead of dense layer, the DCDNN-based autoencoder preserved the position information which can be used as the input of various types of classifier for later prediction.

Table S1. Structure of the *DCDNN*-based autoencoder

type	kernel size / stride (units)	input size
	normal distributed noise	
convolution	1 x 3 / 1 (32)	1 x 23 x 8
	batch normalization	
	normal distributed noise	
convolution	1 x 3 / 2 (64)	1 x 23 x 32
	batch normalization	
	normal distributed noise	
convolution	1 x 3 / 1 (64)	1 x 12 x 64
	batch normalization	
	normal distributed noise	
convolution	1 x 3 / 2 (256)	1 x 12 x 64
	batch normalization	
	normal distributed noise	
convolution	1 x 3 / 1 (256)	1 x 6 x 256
	batch normalization	
	normal distributed noise	
deconvolution	1 x 3 / 2 (256)	1 x 6 x 256
	batch normalization	
deconvolution	1 x 3 / 1 (256)	1 x 12 x 256
	batch normalization	
deconvolution	1 x 3 / 2 (64)	1 x 12 x 256
	batch normalization	
deconvolution	1 x 3 / 1 (64)	1 x 24 x 64
	batch normalization	
deconvolution	1 x 3 / 2* (32)	1 x 24 x 64
	batch normalization	
deconvolution	1 x 3 / 1 (8)	1 x 23 x 32

For on-target and off-target prediction, we established two convolutional neural networks with pre-training based fine-tuning of the encoder part of the *DCDNN*-based autoencoder described before. The structure of both networks are shown in Table S2 for on-target prediction model and Table S3 for off-target prediction model respectively. In our model, we only used convolution layer for feature extraction and prediction. Because convolution layer has fewer parameters compared with dense layer, also it can process information channel-wisely instead of mixing all the information regardless of its location. Also, we used stride-two convolution layer to replace the pooling layers used by common convolution neural network, since not only can it reduce the dimension of the input just like a pooling layer but also perform convolution operation to extract high-order features which could be more effective and information-preserved. On the other hand, we only used batch normalization instead of other regularization methods since batch normalization can cope with both the underfitting and overfitting problem in a robust way. This setup can lead to better a feature extraction with higher efficiency and computational complexity as well as better generalization ability.

Our networks were trained with the *TensorFlow* framework on two NVIDIA GTX 1080Ti GPUs. The models presented were achieved using the *Adam optimizer* with initial learning rate of 0.001, beta1 of 0.9, beta2 of 0.999 and epsilon of 1e-8. The optimized parameters will be tuned automatically under the *Adam optimizer*.

Table S2. Structure of the *CNN*-based on-target prediction model

type	kernel size / stride (units)	input size
convolution	1 x 3 / 1 (32)	1 x 23 x 8
batch normalization		
convolution	1 x 3 / 2 (64)	1 x 23 x 32
batch normalization		
convolution	1 x 3 / 1 (64)	1 x 12 x 64
batch normalization		
convolution	1 x 3 / 2 (256)	1 x 12 x 64
batch normalization		
convolution	1 x 3 / 1 (256)	1 x 6 x 256
batch normalization		
convolution	1 x 3 / 2 (512)	1 x 6 x 256
batch normalization		
convolution	1 x 3 / 1 (512)	1 x 3 x 512
batch normalization		
convolution	1 x 3 / 1 (1024)	1 x 3 x 512
batch normalization		
convolution	1 x 1 / 1 (2)	1 x 1 x 1024
softmax (1 x 1 x 2)/identity		

Table S3. Structure of the *CNN*-based off-target prediction model

type	kernel size / stride (units)	input size	number
convolution	1 x 3 / 1 (32)	1 x 23 x 8	2
batch normalization			2
convolution	1 x 3 / 2 (64)	1 x 23 x 32	2
batch normalization			2
convolution	1 x 3 / 1 (64)	1 x 12 x 64	2
batch normalization			2
convolution	1 x 3 / 2 (256)	1 x 12 x 64	2
batch normalization			2
convolution	1 x 3 / 1 (256)	1 x 6 x 256	2
batch normalization			2
channel-wise concatenation			1
convolution	1 x 3 / 2 (512)	1 x 6 x 512	1
batch normalization			1
convolution	1 x 3 / 1 (512)	1 x 3 x 512	1
batch normalization			1
convolution	1 x 3 / 1 (1024)	1 x 3 x 512	1
batch normalization			1
convolution	1 x 1 / 1 (2)	1 x 1 x 1024	1
softmax (1 x 1 x 2)/identity			1

Detailed source code is available at https://github.com/bm2-lab/DeepCRISPR
